# Does Practitioner Experience Affect Intraocular Pressure After Endotracheal Intubation?

**DOI:** 10.7759/cureus.36593

**Published:** 2023-03-23

**Authors:** Ceyda Baskan, Semih Baskan

**Affiliations:** 1 Ophthalmology, Ankara Bilkent City Hospital, Ankara, TUR; 2 Anesthesiology and Reanimation, Ankara Bilkent City Hospital, Ankara, TUR

**Keywords:** resident doctor, tracheal intubation, intubation skill, hemodynamic parameters, intraocular pressure

## Abstract

Aim: Intraocular pressure (IOP) may decrease or increase during general anesthesia, depending on various factors. This study aimed to investigate the effects of the level of provider training period on post-intubation IOP values and hemodynamic response.

Methods: This study was a cross-sectional observational study. Before inclusion in the study, informed consent was obtained from all participants. The study was approved by the local ethical committee. The study included 120 adult patients, both sexes, aged between 18 and 65, with physical statuses according to the American Society of Anesthesiologists (ASA) I or II, Mallampati score I. The research included 120 anesthesiologist resident doctors who received their training in our clinic. In this study, anesthesiology resident doctors were divided into three separate seniority groups (group 1, less than one-year residents in anesthesiology who had performed fewer than 10 intubations; group 2, one- to three-year residents; and group 3, more than three-year residents). After receiving a standard intravenous induction, direct laryngoscopy and endotracheal intubation techniques were performed. Systolic blood pressure (SBP), diastolic blood pressure (DBP), heart rate (HR), and IOP were measured and recorded before pre-induction (T1), the first minute after induction (T2), and the first minute after laryngoscopy and intubation (T3).

Results: There was no statistically significant difference (p > 0.05) between groups in the values of IOP, SBP, DBP, and HR measured at T1, T2, and T3. Measurements at T1, T2, and T3 were similar in all three groups. Comparisons within the groups revealed that IOP values at all measurement times (T1, T2, and T3) were different from each other in less than three-year resident groups. This difference was statistically significant (p < 0.001). The measurement values at T2 were the lowest and T3 were the highest in less than three-year resident groups. There was a significant increase in IOP after endotracheal intubation (T3) compared to baseline levels (T1) in less than three-year resident groups. IOP values at T2 were also significantly lower than the values at T1 and T3 (p < 0.001) in the more than three-year resident group (group 3). However, when we compared IOP measurements at T1 and T3 among themselves in the more than three-year resident group, no significant difference was found (p > 0.05).

Conclusion: This study showed that endotracheal intubation in general anesthesia practice is performed most effectively by resident doctors with more than three years of anesthesiology training, without changing the IOP value.

## Introduction

Intraocular pressure (IOP), which is the pressure of the eye contents exerted on its enclosing wall, is crucial for maintaining the eye's refractive qualities. IOP is more important than blood pressure (BP) in determining retinal function at increased levels since it linearly lowers the perfusion of the ocular tissues. Raised IOP induces compression of the vasculature at a pressure higher than the ocular perfusion pressure (OPP), which in animal models results in retinal ischemia and blindness. In healthy individuals, an acute IOP increase of around 20 mmHg for five minutes lowers blood flow to the retina, choroid, and optic nerve and may impair the transfer of vital neurotrophins from the brain to the retina. A sudden drop in IOP (6.5 mmHg) can lead to hypotonic maculopathy, which impairs vision and may result in retinal detachment. Acute elevations in IOP in an "open" globe, such as after retinal surgery, may cause expulsion of the contents of the orbit or expulsive hemorrhage. Due to circadian control of aqueous humor secretion, the normal IOP is 16 ± 5 mmHg. Pathologic values are those that are more than 24 mmHg [1].

IOP may decrease or increase during general anesthesia, depending on various factors such as the type and dose of anesthetic agent, the patient's age and medical history, and the surgical procedure. Some anesthetic agents can cause a decrease in IOP due to vasodilation and decreased production of aqueous humor. On the other hand, certain anesthetic agents and surgical positions can increase IOP [2].

Laryngoscopy and tracheal intubation can cause increases in BP, heart rate (HR), and IOP due to stimulation of the autonomic nervous system and the release of stress hormones such as adrenaline. These changes are generally considered normal physiological responses [3,4].

The effect of an anesthesiologist resident doctor’s training period on post-intubation IOP values and the hemodynamic response has not yet been studied. Anesthesiologist resident doctors, who are certified and trained in anesthesia, are increasingly becoming involved in the care of patients during intubation. Their involvement may help improve patient outcomes and reduce the risks associated with intubation.

The aim of this study is to investigate the effect of an anesthesiologist resident doctor’s training period on post-intubation IOP values and hemodynamic response.

## Materials and methods

After receiving clearance from the hospital's ethical committee, the study was carried out at the anesthesiology and reanimation department of an education and research hospital, Ankara Bilkent City Hospital . The study was conducted in accordance with the Declaration of Helsinki and approved by the Institutional Ethics Committee of Ankara Bilkent City Hospital (IRB number -E2-22-2886). Before inclusion in the study, informed consent was obtained from all participants. The study included 120 adult patients, both sexes, aged between 18 and 65, with physical statuses of Grade I or II according to the American Society of Anesthesiologists (ASA), Mallampati score I; who have a body mass index (BMI) of <30 kg/m2; who are scheduled for elective surgery (62 patients (51.7%) had a septoplasty, 30 patients (25%) had a laparoscopic cholecystectomy, and 28 patients (23.3%) had a rhinoplasty) under general anesthesia; and who require endotracheal intubation between December 2022 and January 2023. Patients under general anesthesia who underwent successful endotracheal intubation in a single attempt were included in the study.

Due to the potential impact on IOP, patients with hypertension, cardiovascular illness, eye disease, vasoactive medication use, glaucoma, ophthalmic medication use, and airway issues were excluded from the research.

Standard monitors (noninvasive BP, electrocardiography, and pulse oximetry) and an intravenous line were connected in the operating room.

The research included 120 of the 142 anesthesiologist resident doctors who received their training in our clinic (22 anesthesiologist resident doctors were excluded due to personal choice or extenuating circumstances). Volunteers were assigned to the study at random using the internet order at www.random.org based on their application number for intubation in the clinical trial list.

After receiving a balanced intravenous induction with lidocaine, propofol, remifentanil, and rocuronium (lidocaine HCL (2%, 20 mg/ml) 1.5 mg/kg, propofol (2%, 20 mg/ml) 1.5 mg/kg, remifentanil (2 mg) 1 μg/kg, and rocuronium (10 mg/ml) 0.6 mg/kg administration intravenously), direct laryngoscopy and endotracheal intubation techniques were performed.

In this study, anesthesiology resident doctors were divided into three separate seniority groups, as suggested by the Turkish Medical Specialization Board in the core training program. Group 1, less than one-year residents in anesthesiology who had performed fewer than 10 intubations; group 2, one- to three-year residents; and group 3, more than three-year residents were included in the study. The residents' training period and patient demographic information were recorded. Endotracheal intubation with a proper-sized high-volume, low-pressure, cuffed endotracheal tube was attempted. IOP and the hemodynamic response parameters (systolic BP (SBP), diastolic BP (DBP), and HR) were measured and recorded before pre-induction (T1), the first minute after induction (T2), and the first minute after laryngoscopy and intubation (T3). IOP measurements were taken using a Tono-Pen AVIA (Reichert, Buffalo, NY) hand-held tonometer on the left eye of the patients after the installation of topical anesthetic with proparacaine hydrochloride 0.5% solution at T1, T2, and T3. IOP was recorded as the primary outcome parameter. Hemodynamic measurements were recorded as the secondary outcome parameter.

Statistical analysis

Data analysis was performed using IBM SPSS Statistics, version 25.0 (IBM Corp., Armonk, NY). While evaluating the study data, the chi-square (χ²) test was used to compare qualitative data as well as descriptive statistical methods (frequency, percentage, mean, standard deviation, median, min-max, skewness, and kurtosis) and graphical methods (histogram, Q-Q plot, stem and leaf plot, and boxplot). The one-way analysis of variance (ANOVA) test was used for comparisons of normally distributed quantitative data between groups, and the repeated measures ANOVA test was used for in-group comparisons. Tukey or Bonferroni post hoc (multiple comparisons) tests were used to find the source. Relationships between variables were evaluated with the Pearson correlation test. The statistical significance level was accepted as p < 0.05.

## Results

There was no statistically significant difference (p > 0.05) between the groups in the values of IOP, SBP, DBP, and HR measured at T1, T2, and T3. Measurements at T1, T2, and T3 were similar in all three groups.

When the comparisons were done within each group, it was seen that there were significant differences in less than three-year resident groups (groups 1 and 2). Comparisons within the groups revealed that IOP values at all measurement times (T1, T2, and T3) were different from each other in less than three-year resident groups. This difference was statistically significant (p < 0.001). The measurement values at T2 were the lowest and T3 were the highest in less than three-year resident groups. This meant that there was a significant increase in IOP after endotracheal intubation (T3) compared to baseline levels (T1: pre-induction) in less than three-year resident groups (Table 1).

**Table 1:** Comparison of the IOP and hemodynamic parameters according to the anesthesiologist resident doctor's training period ^a ^chi-square test (n (%)); ^b^ one-way ANOVA test (mean ± SD); c: repeated measures ANOVA test (mean ± SD). BMI, body mass index; ASA, American Society of Anesthesiologists; IOP, intraocular pressure (normal range: 10-24 mmHg); SBP, systolic blood pressure; DBP: diastolic blood pressure; HR: heart rate; T1, pre-induction; T2, the first minute after induction; T3: the first minute after laryngoscopy and intubation

		<1 year (n = 26)	1-3 years (n = 59)	>3 years (n = 35)	p
Resident doctor gender	Female	19 (%73.1)	36 (%61.0)	20 (%57.1)	0.422^a^
	Male	7 (%26.9)	23 (%39.0)	15 (%42.9)	
Patient gender	Female	21 (%80.8)	30 (%50.8)	19 (%54.3)	0.030^a^
	Male	5 (%19.2)	29 (%49.2)	16 (%45.7)	
Age (year)		34.6 ± 11.8	35.1 ± 11.1	35.7 ± 14.5	0.937^b^
Weight (kg)		71.0 ± 16.6	71.0 ± 11.8	74.8 ± 14.4	0.396^b^
Height (cm)		170.2 ± 9.3	169.9 ± 8.9	170.9 ± 9.8	0.882^b^
BMI (kg/m^2^)		24.3 ± 4.2	24.5 ± 3.2	25.6 ± 4.2	0.312^b^
ASA	I	19 (%73.1)	40 (%67.8)	24 (%68.6)	0.885^a^
	II	7 (%26.9)	19 (%32.2)	11 (%31.4)	
Smoking	Absent	21 (%80.8)	42 (%71.2)	30 (%85.7)	0.239^a^
	Present	5 (%19.2)	17 (%28.8)	5 (%14.3)	
IOP (mmHg)	T1	12.5 ± 2.3	13.0 ± 1.9	13.0 ± 2.0	0.574^b^
	T2	11.1 ± 3.1	11.2 ± 2.5	11.7 ± 2.2	0.612^b^
	T3	15.0 ± 3.8	15.6 ± 3.5	14.0 ± 3.5	0.123^b^
	p	<0.001^c^	<0.001^c^	<0.001^c^	
	Difference	1 - 2 - 3	1 - 2 - 3	2 with 1 - 3	
SBP (mmHg)	T1	128.3 ± 16.1	134.4 ± 15.9	140.8 ± 18.6	0.117^b^
	T2	113.2 ± 14.4	108.6 ± 15.3	106.0 ± 13.6	0.169^b^
	T3	122.9 ± 21.5	126.9 ± 22.0	123.1 ± 19.9	0.604^b^
	p	<0.001^c^	<0.001^c^	<0.001^c^	
	Difference	2 with 1 - 3	1 - 2 - 3	1 - 2 - 3	
DBP (mmHg)	T1	75.6 ± 9.0	78.0 ± 12.2	79.0 ± 9.5	0.468^b^
	T2	65.6 ± 11.9	63.7 ± 11.7	59.0 ± 10.4	0.060^b^
	T3	73.5 ± 18.1	78.0 ± 17.5	72.6 ± 16.8	0.282^b^
	p	<0.001^c^	<0.001^c^	<0.001^c^	
	Difference	2 with 1 - 3	2 with 1 - 3	2 with 1 - 3	
HR (beats/min)	T1	80.8 ± 12.3	80.2 ± 13.0	85.5 ± 16.1	0.193^b^
	T2	75.9 ± 10.0	78.7 ± 12.6	78.8 ± 15.3	0.606^b^
	T3	87.8 ± 12.8	88.0 ± 14.4	87.0 ± 15.4	0.947^b^
	p	<0.001^c^	<0.001^c^	<0.001^c^	
	Difference	1 - 2 - 3	3 with 1 - 2	2 with 1 - 3	

IOP values at T2 were also significantly lower than the values at T1 and T3 (p < 0.001) in the more than three-year resident group (group 3). However, when we compared IOP measurements at T1 and T3 among themselves in the more than three-year resident group, no significant difference was found (p > 0.05) (Table 2).

**Table 2:** Comparison of the IOP according to the anesthesiologist resident doctor's training period at measurement times of T1 and T3 ^a ^one-way ANOVA test (mean ± SD); ^b^ paired samples t-test (mean ± SD). IOP, intraocular pressure; T1, pre-induction; T3, the first minute after laryngoscopy and intubation

		<1 year (n = 26)	1-3 years (n = 59)	>3 years (n = 35)	p
IOP	T1	12.5 ± 2.3	13.0 ± 1.9	13.0 ± 2.0	0.574^a^
	T3	15.0 ± 3.8	15.6 ± 3.5	14.0 ± 3.5	0.123^a^
	p	<0.001^b^	<0.001^b^	<0.118^b^	

**Figure 1:**
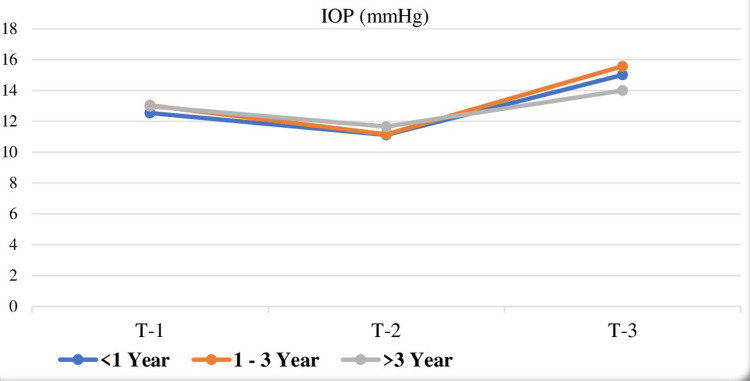
Changes in IOP values according to the anesthesiology resident's training period. IOP values in all measurement times were different from each other in groups 1 (less than one year) and 2 (one to three years) (p < 0.001). IOP values at T2 were significantly lower than the values at T1 and T3 (p < 0.001) in group 3 (more than three years). IOP increase at T3 was not statistically significant in group 3 (repeated measures ANOVA test was used for in-group comparisons, and the statistical significance level was accepted as p < 0.001) T1, pre-induction; T2, the first minute after induction; T3, the first minute after laryngoscopy and intubation

SBP values were different in all measurement times (T1, T2, and T3) in groups 2 and 3. The values at T2 were the lowest and T1 were the highest in both groups. However, in group 1, only the values at T2 were lower than the values at T1 and T3 (p < 0.001). There was a significant increase in SBP at T3 in group 1 (p < 0.001). DBP values were the lowest at T2 in all groups. HR values were the lowest at T2 and the highest at T3 in group 1. There was also a significant increase in HR at T3 in group 2 (p < 0.001). However, this increase at T3 was not demonstrated in group 3, and there was only a significant decrease in HR at T2 (p < 0.001). IOP change at T1, T2, and T3 is illustrated in Figure 1.

**Figure 2:**
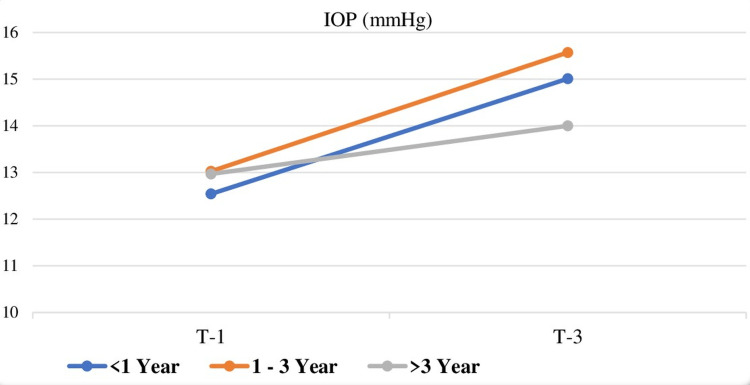
Changes in IOP values according to the anesthesiology resident's training period at T1 and T3. IOP values at T1 and T3 were different from each other in groups 1 (less than one year) and 2 (one to three years) (p < 0.001). There was no significant increase in IOP value at T3 in group 3 (more than three years) (p > 0.05) (repeated measures ANOVA test was used for in-group comparisons, and the statistical significance level was accepted as p < 0.001) T1, pre-induction; T2, the first minute after induction; T3, the first minute after laryngoscopy and intubation

IOP changes at T1 and T3 are illustrated in Figure 2.

## Discussion

In this study, a decrease in IOP values after induction of the anesthesia was detected in all practitioner groups. IOP values increased after endotracheal intubation compared to post-induction values in all seniority groups. However, there was no significant increase in the post-intubation IOP values in the most senior group (more than three-year residents) compared to the baseline values, while there was a significant increase in the other groups (less than three-year residents). These findings showed that the most experienced anesthesia seniority group performed the most appropriate intubation due to the stability of the IOP after endotracheal intubation. An anesthesiologist must complete a five-year residency program in anesthesiology after obtaining a medical degree. During this program, they receive extensive training in the administration of anesthesia, including intubation skills. Their involvement may improve patient outcomes and reduce the risks associated with intubation. The effect of an anesthesiologist resident’s training period on post-intubation IOP values and the hemodynamic response has not yet been studied. This study aimed to investigate the effect of an anesthesiologist resident doctor’s training period on post-intubation IOP values and hemodynamic response. It was aimed to utilize a prospective observational design to compare pre-induction and post-intubation IOP values and hemodynamic parameters among patients who had their intubation procedure performed by anesthesiologist resident doctors with varying levels of training. The results of this study may help to identify the potential benefits of increased anesthesiologist resident doctor’s training on patient outcomes.

Adequate vascular perfusion is crucial to maintaining the functional integrity of the retina, as it is one of the most metabolically active tissues in the body. Morphologically and functionally, two different arterial systems are present to regulate this vascular perfusion. While retinal circulation feeds the inner retina and optic nerve head, choroidal circulation is responsible for the outer layers of the retina [5]. Because the relationship between OPP, mean arterial pressure (MAP), and IOP is complicated and has a remarkable variation between individuals, autoregulation mechanisms of both choroidal and retinal circulations respond to changes in OPP. Engorgement of the choroid results from increased intraocular blood volume and a transient rise in IOP when venous outflow fails [6,7]. Retinal vascular perfusion is dependent on IOP. When IOP exceeds MAP, ocular blood flow stops and results in low OPP. OPP decrement may also be caused by a decrease in MAP [8].

It is known that all frequently applied anesthetic induction drugs reduce IOP, such as propofol and opioids. Opioids are examples of short-acting opioids that considerably lower IOP during anesthesia induction and continue to do so following laryngoscopy [9]. The exact mechanism of this decrease is not fully understood, but it is thought to be related to changes in blood flow and IOP. After the anesthesia wears off, IOP typically returns to its normal level. However, in some cases, the decrease in IOP can be prolonged, leading to a temporary lowering of the patient's IOP. Changes in blood flow and pressure can affect IOP. Anesthesia can cause a decrease in BP, pulse, and IOP as it can cause systemic and local vasodilation, leading to decreased vascular resistance. This effect can vary depending on the type of anesthesia and the patient's medical history. The most notable increase in IOP under general anesthesia happens during laryngoscopy. Tracheal intubation can cause an increase in intracranial pressure, which can lead to an increase in IOP. However, it is important to note that the effect of IOP can vary greatly between patients and depends on several factors, such as the individual's anatomy and the method of intubation [1,2,10,11].

Increased IOP can be dangerous in certain eye surgeries, such as glaucoma filtering, cataract, retinal detachment, and refractive surgeries [12,13]. The minimum level of IOP change in eye surgeries facilitates the work of surgeons and reduces complications. If possible, it is always desirable for this change to be low during general anesthesia. Practitioner experience can impact IOP after endotracheal intubation due to several factors, such as proper technique and experience in recognizing and addressing potential complications during the procedure [10].

In our study, as compatible with other studies, IOP values were found to be low after induction and high after intubation compared to pre-induction values in less than three-year assistant groups [1,2,10,11]. This increase in IOP is within normal limits. There was not any pathological increase. However, in our study, there was not any significant increase in IOP values after intubation in residents over three years compared to the initial values. Therefore, as experience increased, effective endotracheal intubation has been performed.

The length of anesthesia training may affect the hemodynamic response during endotracheal intubation. Singhal et al. [14] have shown that longer training periods result in decreased hemodynamic responses during intubation procedures. This suggests that the more experience an anesthesia provider has, the better they can control the hemodynamic response during endotracheal intubation.

Anesthesiologists use a combination of techniques to control BP and maintain stable IOP. These techniques include the use of medications to lower and carefully monitor BP and HR and control fluid and electrolyte balance [15]. Maintaining stable IOP during ophthalmology surgeries requires careful monitoring and management throughout the procedure as well as close collaboration between the anesthesiologist and surgical team. Our study showed that as experience increased, undesirable changes due to anesthesia decreased. However, despite all these results, after effective endotracheal intubation and subsequent anesthesia, IOP and hemodynamic changes remain within normal limits. The increases or decreases observed in this study were within normal limits. Therefore, achieving effective endotracheal intubation may be more important.

The limitation of our study is that, because of considering patient safety, we included patients who had uneventful endotracheal intubation on the first attempt. Therefore, we could not evaluate the effects of endotracheal intubation on hemodynamic response and IOP after repeated attempts.

## Conclusions

This study showed that endotracheal intubation in general anesthesia practice is performed most effectively by resident doctors with more than three years of anesthesiology training, without changing the IOP value. Controlling IOP during general anesthesia is a critical component of ensuring the success of ophthalmic surgeries and requires the expertise of trained and experienced anesthesiologists. Due to the delicate nature of eye surgeries, the absence of IOP and hemodynamic changes during the surgery is highly desirable.
